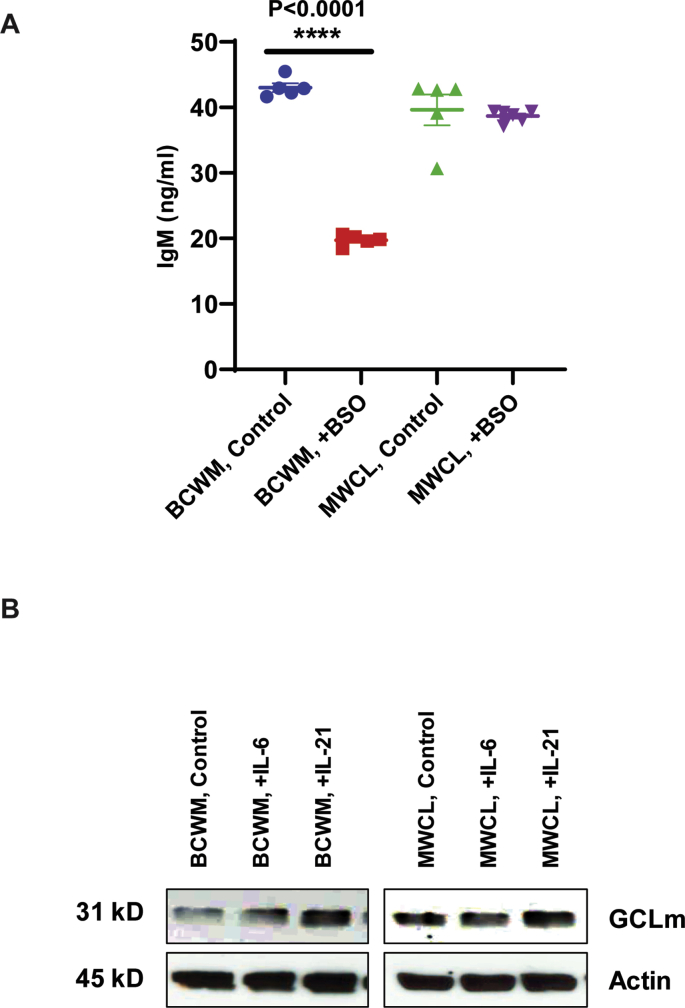# Increased glutathione utilization augments tumor cell proliferation in Waldenstrom Macroglobulinemia

**DOI:** 10.1016/j.redox.2020.101657

**Published:** 2020-08-01

**Authors:** Shahrzad Jalali, Jie Shi, Alex Buko, Nagib Ahsan, Jonas Paludo, Makayla Serres, Linda E. Wellik, Jithma Abeykoon, HyoJin Kim, Xinyi Tang, Zhi-Zhang Yang, Anne J. Novak, Thomas E. Witzig, Stephen M. Ansell

**Affiliations:** a Division of Hematology and Internal Medicine, Mayo Clinic, Rochester, MN, USA; b Department of Hematology, Henan Provincial People's Hospital, People's Hospital of Zhengzhou University, Zhengzhou, Henan, China; c Human Metabolome Technologies (HMT) America, Boston, MA, USA; d COBRE Center for Cancer Research Development, Proteomics Core Facility, Rhode Island Hospital, Providence, RI, 02903, USA; e Division of Biology and Medicine, Brown University, Providence, RI, 02903, USA

**Keywords:** Waldenstrom Macroglobulinemia, Metabolism, Glutathione

## Abstract

Metabolic reprogramming is a hallmark of cancer cells. In Waldenstrom Macroglobulinemia (WM), the infiltration of IgM-secreting lymphoplamacytic cells into the bone marrow (BM) could shift the homeostasis of proteins and metabolites towards a permissive niche for tumor growth. Here, we investigated whether alerted metabolic pathways contribute to the pathobiology of WM and whether the cytokine composition of the BM promotes such changes.

Metabolomics analysis on WM patients and normal donors’ serum samples revealed a total of 75 metabolites that were significantly altered between two groups. While these metabolites belonged to amino acids, glucose, glutathione and lipid metabolism pathways, the highest number of the differentially expressed metabolites belonged to glutathione metabolism. Proteomics analysis and immunohistochemical staining both confirmed the increased protein levels mediating glutathione metabolism, including GCLC, MT1X, QPCT and GPX3. Moreover, treatment with IL-6 and IL-21, cytokines that induce WM cell proliferation and IgM secretion, increased gene expression of the amino acid transporters mediating glutathione metabolism, including ASCT2, SLC7A11 and 4F2HC, indicating that cytokines in the WM BM could modulate glutathione metabolism. Glutathione synthesis inhibition using Buthionine sulphoximine (BSO) significantly reduced WM cells proliferation *in vitro*, accompanied with decreased NFκB-p65 and MAPK-p38 phosphorylation. Moreover, BSO treatment significantly reduced the tumor growth rate in a WM xenograft model, further highlighting the role of glutathione metabolism in promoting tumor growth and proliferation.

In summary, our data highlight a central role for glutathione metabolism in WM pathobiology and indicate that intervening with the metabolic processes could be a potential therapy for WM patients.

## 1 Introduction

The metabolic reprogramming of cancer cells provides them with energy and building blocks, adapts them to unfavorable microenvironmental stress and gives rise to drug resistance. Therefore, the delineation of metabolic patterns associated with certain cancers could potentially offer diagnostic, prognostic and therapeutic benefits. The significance of certain metabolic pathways in promoting cell proliferation has previously been investigated in several hematological malignancies. For instance, myeloid leukemia cells are highly dependent on glucose metabolism and glycogen synthesis for their proliferation [1]. Moreover, the driver mutations affecting isocitrate dehydrogenase (IDH)-1 and −2 genes are shown to result in accumulation of oncometabolite 2-hydroxyglutarate that has a prognostic value in acute myeloid leukemia (AML) [2,3]. Metabolomics analysis of a large cohort of AML patients’ serum samples has also identified a panel of 6 glucose-related metabolite markers that have prognostic value in the cytogenetically normal AML patients [4], validating the significance of glycolysis and TCA cycle in this cancer.

Non-targeted metabolomics analysis of the serum samples in multiple myeloma (MM) has identified significantly altered metabolites that differentiate patients with advanced disease from normal samples [5]. In another study, metabolomics analysis has shown the altered levels of some metabolites, including glutamine, lysine and cholesterol, at the time of diagnosis that are returned back to normal levels upon achieving complete remission, suggesting these metabolites as the potential biomarkers of response to the therapy [6]. Similarly, the metabolism of cancer cells has also been studied in non-Hodgkin B-cell lymphomas. Higher level of hexokinase II (HK-II) is shown to be associated with shorter progression free survival (PFS) or overall survival (OS) in the patients with diffuse large B-cell lymphoma (DLBCL) [7].

Given the pivotal role of metabolism reprograming in cancer cell biology, inhibition of the molecules involved in glucose, glutamine or fatty acid oxidation metabolism has shown promising results in suppressing tumor cell proliferation in several blood cancers [[8], [9], [10], [11]]. However, despite such available reports, to date the implications of metabolic pathways in tumor progression of Waldenstrom Macroglobulinemia (WM), an indolent B-cell lymphoma, remain largely unknown. WM is mainly characterized by increased infiltration and expansion of monoclonal immunoglobulin M (IgM)-secreting lymphoplasmacytic lymphoma (LPL) cells within the bone marrow (BM) microenvironment [12]. Whole genome sequencing analysis has identified activating mutations in myeloid differentiation factor 88 (MyD88^mut^), CXCR4^WHIM^ and ARID1A as the most prevalent mutations in WM, comprising more than 90%, 30% and 17% of the patients respectively [13]. Activating mutation in MyD88 is shown to induce survival NFκB signaling pathway via IRAK1/IRAK4 or bruton tyrosine kinase (BTK) pathways and also increase transcription and activation of SRC family member, HCK, which in turn increases WM tumor cell growth and proliferation via BTK, AKT and ERK signaling pathways [14]. Activating mutation of CXCR4 increases the recruitment of WM cells to the BM via interaction with SDF-1 (CXCR12) on the surface of the BM stromal cells [15]. Currently, rituximab-, proteasome inhibitor- or BTK inhibitor-based therapies are widely used for treating patients with symptomatic WM, but limitations have been reported using these therapeutic agents [16]. Patients with wild type MYD88 (MyD88^wt^) have shorter median OS in response to ibrutinib treatment than MyD88^mut^ [17]. Moreover, the presence of CXCR4^WHIM^ mutation has been shown to be associated with an adverse therapeutic response to ibrutinib [18]. Generally, genetic heterogeneity of the malignant cells, the development of resistance to the treatment agents and the toxicities associated with the novel agents, generate a mixed response rates so that some patients show refractory or recurrence after therapy. Therefore, it is crucial to identify novel therapeutic targets in WM.

Here, we have employed metabolomics and proteomics technologies in order to identify the key metabolic pathways that drive WM tumor growth and progression. We have further validated our results in both *in vitro* and *in vivo* models using established WM cell lines.

## 2 Materials and methods

### 2.1 Patients’ specimens

All samples, including peripheral blood serum and BM plasma, were received from the WM patients and normal subjects under Mayo Clinic institutional review board approval. We obtained the normal serum samples from age-matched patients included in the Mayo Clinic biobank. Normal serum samples were from both males (n = 12) and females (n = 10), with a median age of 66.5 years. Similarly, WM patient's samples were from both males and females (11 males, 7 females), with a median age of 64 years. BM plasma samples were the fractionation products of BM aspirates which included soluble non-cellular components of the patients BM aspirates. WM patients involved those with progressing disease, including both untreated and post treatment patients. Control BM plasma specimens were collected from the patients who had undergone hip replacement surgery.

### 2.2 Metabolome analysis

Analysis of the metabolites in the human BM plasma and serum samples was performed using Capillary Electrophoresis Time-of-Flight Mass Spectrometry (CE-TOFMS) and Liquid Chromatography Time-of-Flight Mass Spectrometry (LC-TOFMS) in positive and negative modes for cationic and anionic metabolites on the basis of Human Metabolome Technologies (HMTs) standard library (Supplementary materials and methods).

### 2.3 Proteomics analysis

Serum samples (22 normal and 41 WM) were subjected to label-free quantitative proteomic analysis according to the procedures explained in detail in the ‘Supplementary Materials and Methods’.

### 2.4 Cell culture and reagents

Two WM cell lines BCWM.1 (a gift from Dr. Steven Treon) and BCWM.1, established in our laboratory [19], were used in this study. Cells were maintained in RPMI 1640 (Invitrogen) supplemented with 50 U/mL penicillin G, 10 μg/mL streptomycin, 10% heat-inactivated fetal bovine serum (FBS), 1 mM sodium pyruvate and 1% MEM non-essential amino acids at 37 °C with 5% CO2. Recombinant human IL-6 and IL-21 were obtained from PeproTech.

### 2.5 Immunohistochemistry

Formalin-Fixed Paraffin-Embedded (FFPE) BM biopsy sections from control and WM patients were used for GPX3 staining. Following deparaffinization, blocking of endogenous peroxidase and antigen retrieval, slides were sequentially incubated with glutathione peroxidase-3 (GPX-3) primary antibody (Abcam) for 30 min, rabbit anti-goat linker antibody for 30 min, rabbit probe for 20 min and R-polymer HRP for 20 min, using MACH 3™ rabbit or mouse-probe HRP Polymer Kit (Biocare Medical). Slides were then incubated with chromogen DAB+ (DakoCytomation) and then counterstained in Meyer's hematoxylin. All slides were scanned using Olympus Ax70 microscope.

### 2.6 siRNA transfection

MWCL-1 cells were transfected with 200 nM scrambled or GPX3 siRNA using Amaxa® Human B Cell Nucleofector® Kit (Lonza) and Nucleofector™ 2b Device (program T-030). Cells were then transferred to the complete RPMI media and incubated for 48 h. The transfection efficiency was measured by RT-PCR analysis and viable cells were detected by staining with a live/death cell staining solution and subsequently flow cytometry analysis.

### 2.7 Flow cytometry

To detect cell viability, scrambled or GPX3 siRNA treated cells were stained with fixable viability dye eFlourTM 780 (eBiosciences) and then analyzed on a BECTON DICKINSON (BD) FACSCANTO II and data were processed by FlowJo software (V10.4).

### 2.8 Proliferation assay

Cell proliferation was assessed using 3H-Thymidine ([3H]TdR) incorporation in response to BSO treatment. Briefly, WM cells were seeded at 1000–2000 cells/100 μl density in a 96-well plate and treated with 100 μg/ml BSO for 72 h. Cultured cells were then labeled with 1μCi/well of [3H]TdR for 18 h, harvested and incorporation of [3H]TdR was measured using MicroBeta scintillation counter (PerkinElmer).

### 2.9 Animal xenografts

Animal studies were carried out under the protocols approved by Mayo Clinic Animal Care and Use Committee (IACUC). 4-6 week-old NSG mice were implanted with 4x10^6^ MWCL-1 cells in their flank. Four days post tumor implantation, mice were administered with 20 mM l-buthionine (S,R)-sulfoximine (BSO), an inhibitor of glutathione synthesis, via drinking water. Control mice did not receive the medicated water. Upon visible tumor onset, the volume of tumors was measured twice/week and the tumor growth rate was graphed.

For additional detailed experimental methods refer to ‘Supplementary Materials and Methods’.

### 2.10 Statistical analysis

Unpaired *t*-test analysis was used to compare differences between control untreated and treated samples. Wilcoxon paired non-parametric test was used to determine the significant differences between mean values of patients and normal groups.

## 3 Results

### 3.1 Metabolomics analysis identifies the differentially expressed metabolites in the WM and normal serum samples

**Fig. 1:**
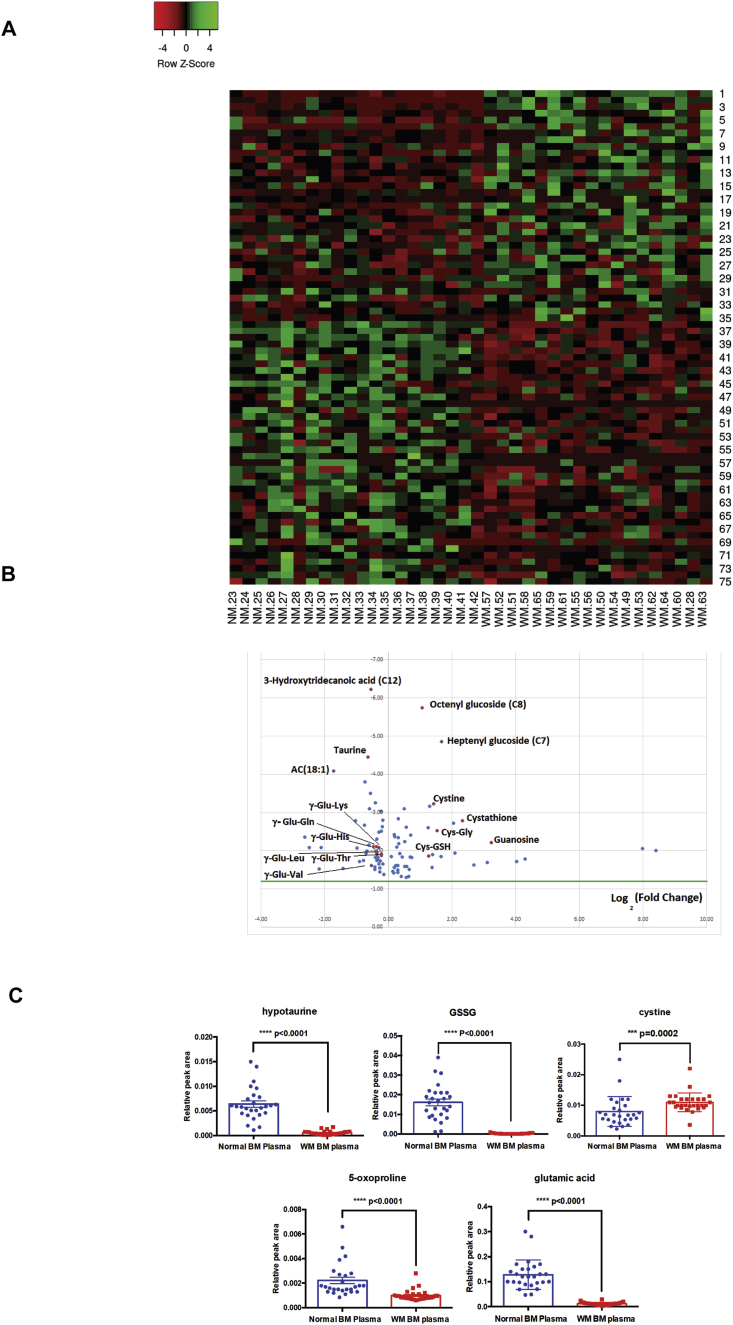
Metabolomic signature of WM patient serum samples as compared to serum from normal donors. A) Heat map analysis showing the differential expression of the metabolites in the WM as compared to normal serum samples. **B)** Volcano plot of non-WM control serum and WM serum samples based on untargeted metabolomics analysis. Y axis: log 10 (0.05), X-axis: Log 2 (10). **C)** The comparison of the metabolite levels, those that are relevant to glutathione pathway, in the normal and WM BM plasma samples.

To investigate the global metabolism changes in WM, serum samples from normal donors (n = 20) and WM (n = 18) patients with progressing disease (including both untreated and post treatment patients) were utilized for untargeted metabolomics analysis. Our data identified a total of 75 metabolites (35 up and 40 down in WM samples) that were differentially expressed between two groups (Fig. 1A and Fig. 1B). Pathway analysis identified that the highest number of the significantly altered metabolites belonged to glutathione metabolism. These metabolites included a wide range of dipeptides composed of gamma (ϒ)-glutamyl-amino acids that were found to be significantly lower in the serum of the WM patients (p < 0.05). In contrast, levels of cysteinyl glycine (Cys-Gly) disulfide, cysteine glutathione disulfide (Cys-GSH), cysteine and the metabolites of ϒ-glutamyl cycle that are produced in response to oxidative stress, were significantly (p < 0.05) higher in the serum of WM patients (Fig. 1A and Fig. 1B & Table 1) (Supplementary Table- 1).

**Table 1:**
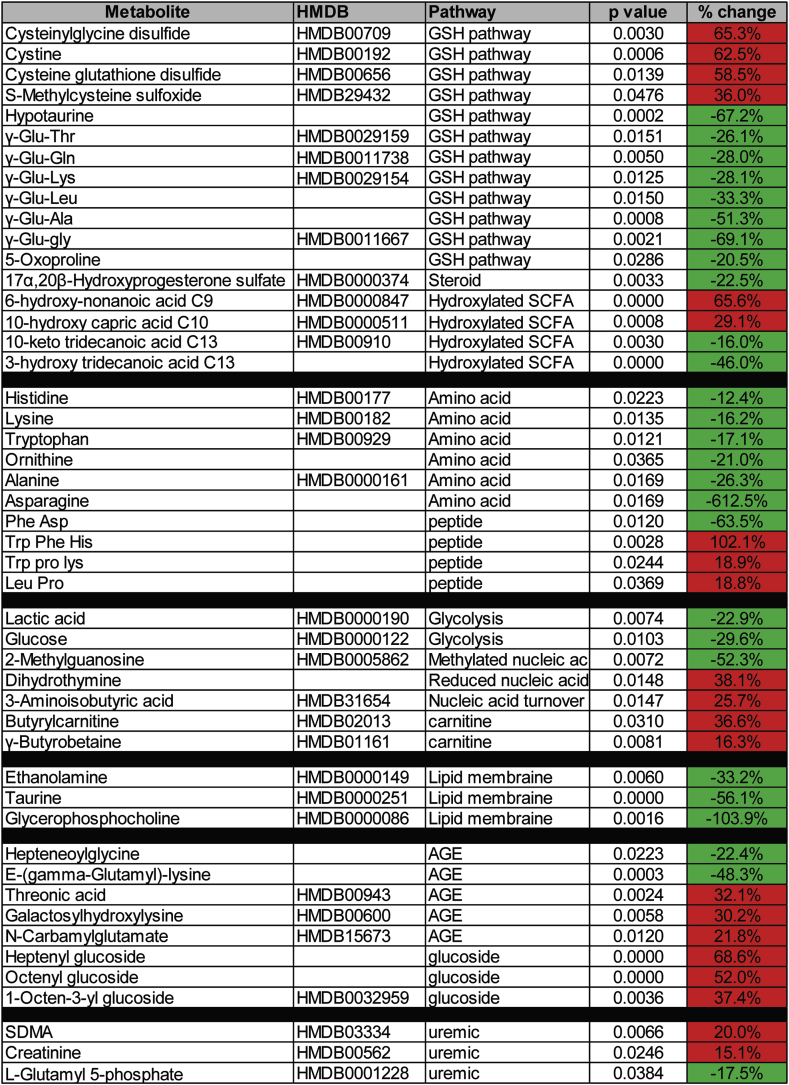
Pathway analysis showing the list of the significantly (p < 0.05) changed metabolites as well as the relevant pathways in the WM serum samples compared to normal serum. HMDB: Human metabolome database.

Glutathione is composed of glycine, cysteine and glutamate amino acids. In the process of glutathione synthesis, ϒ-glutamyl-amino acids serve as precursors for 5-oxoproline and glutamate synthesis, the latter which is then used for the synthesis of glutathione. Therefore, the reduced levels of these metabolites could be an indication of their utilization for glutathione synthesis. Meanwhile, increased levels of the metabolites of ϒ-glutamyl cycle highlights a potential defensive mechanism against the oxidative stress condition generated by the growth of WM cancer cells.

Glutathione pathway metabolites are changed in the BM plasma of the WM patients compared to their normal counterparts.

The main feature of WM is the slow proliferation associated with reduced cell death of malignant lymphoma cells within the BM. This could introduce metabolic stress in the BM and consequently affect the behavior of both malignant and adjacent BM cells. Alterations in the metabolites of glutathione pathway in the WM serum prompted us to also examine changes in the BM plasma in relation to glutathione metabolism. We found that cystine level was higher, whereas hypotaurine, 5-oxoproline, glutathione and glutamic acid levels, the metabolites involved in glutathione metabolism, were lower in the BM plasma of the WM patients than the normal samples (Fig. 1C). Therefore we concluded that similar to serum samples, the metabolites belonging to glutathione metabolism are also altered in the WM BM plasma samples.

Label-free quantitative proteomics analysis reveals increased expression of the enzymes involved in glutathione and reactive-oxygen metabolism in WM patients’ serum samples.

**Fig. 2:**
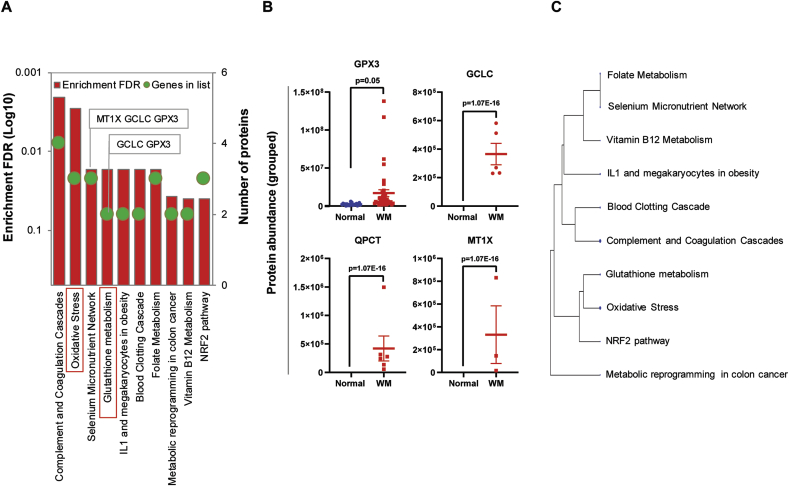
Gene enrichment pathway analysis of the significantly increased proteins in the WM samples compared to the normal serum samples. A) A label-free quantitative proteomics analysis was performed in the WM (n = 41) and normal (n = 22) serum samples followed by pathway analysis on the significantly increased proteins. Bar graphs show the top ten most enriched wiki pathways (data base of biological pathways) that are significantly increased in WM samples. Green circles indicate the number of proteins identified in each pathway. **B)** Comparative protein abundance of the key proteins involved in glutathione and oxidative stress pathways. glutaminyl-peptide cyclotransferase (QPCT), glutathione peroxidase-(GPX)-3, glutamate-cysteine ligase catalytic subunit (GCLC) and Metallothionein-1X (MT1X). **C)** Dendogram represents the tree relationship among ten most enriched wiki pathways. Size of the blue circles indicates the value of enrichment (false discovery rate [FDR]). The Analysis was conducted with an open source bioinformatics platform named ShinyGO (http://bioinformatics.sdstate.edu/go/). (For interpretation of the references to colour in this figure legend, the reader is referred to the Web version of this article.)

The difference in the metabolite levels of glutathione pathway could originate from altered expression and/or activity of the enzymes involved in glutathione synthesis in WM patients. Therefore, we performed label-free quantitative proteomics analysis on WM patients (n = 41) and normal donors (n = 22) serum samples, in order to demonstrate the global proteome expression profile in WM disease. Our data showed a total of 139 proteins were significantly (1.5 fold, p = 0.05) increased in abundance in WM samples compared to the normals (Supplementary Table- 2). Pathway analysis revealed oxidative stress, glutathione metabolism and NRF2 pathways among the most significantly enriched pathways in WM serum samples (Fig. 2A and Fig. 2C), indicating that WM disease is accompanied with high oxidative stress condition. Quantitative proteomic data showed proteins associated with glutathione and oxidative stress metabolism, including glutaminyl-peptide cyclotransferase (QPCT), glutathione peroxidase-(GPX)-3, glutamate-cysteine ligase catalytic subunit (GCLC) and metallothionein-1X (MT1X) were significantly increased, ranging from 3 to >100 fold, in the WM patients, compared to the normal serum samples (Fig. 2B). The metabolic reactions catalyzed by QPCT, GCLC and GPX3 are all key in the cycle of glutathione synthesis. Thus, proteomic results are in accordance with the data obtained from metabolomics analysis, highlighting increased activation of glutathione metabolism in patients with WM.

### 3.2 Total GPX expression and activity is increased in WM samples

**Fig. 3:**
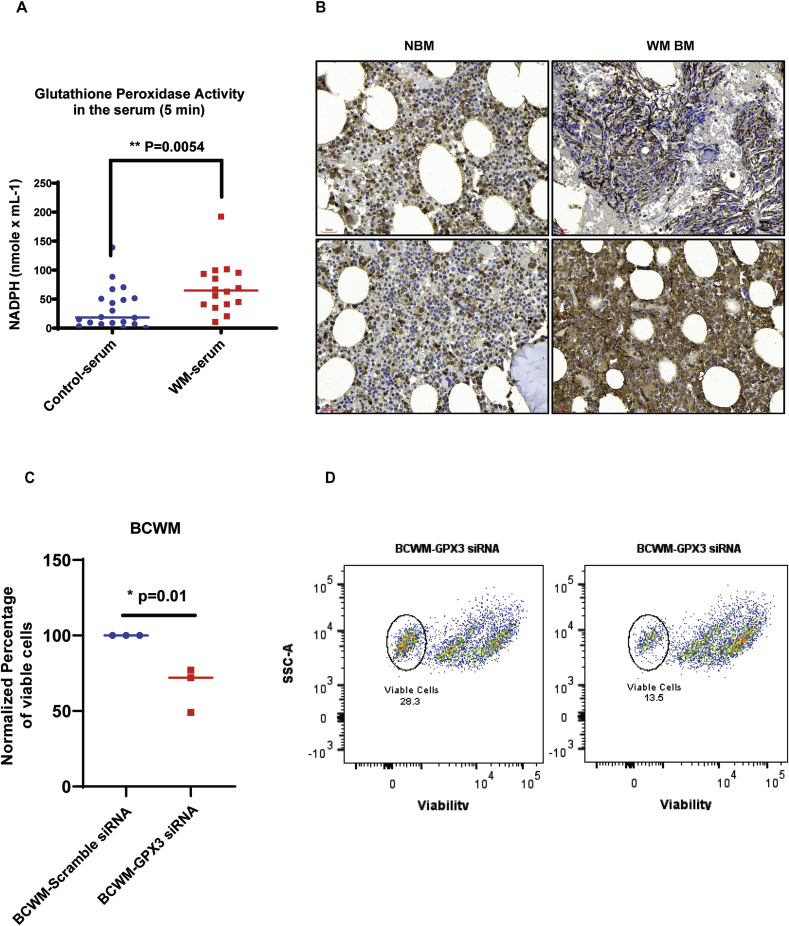
Increased glutathione peroxidase expression and activity in the WM samples, as compared to the normal equivalents. A) Bar graph compares the total GPX activity in the serum of the WM patients (n = 16) and normal donors (n = 20). **B)** IHC analysis of the BM tissue samples from WM patients and normal donors shows the pattern of GPX3 staining. Upper panel and Lower panel belong to two different WM and Normal BM samples. **C)** Dot plot represents normalized viable BCWM.1 cells transfected with either scrambled or GPX3 siRNA (n = 3). **D)** Flow cytometry analysis of the scrambled or GPX3 siRNA transfected BCWM.1 cell lines showing reduced cell viability following GPX3 siRNA transfection. The figure is the representative of three independent experiments.

We measured the total glutathione peroxidase activity in both WM and normal serum in order to investigate whether increased expression of GPX3, identified by proteomics analysis, is also associated with elevated GPX activity. Total GPX activity was found to be significantly higher in WM serum than normal serum specimens (p = 0.0054) (Fig. 3A). We also stained the BM tissue sections to detect GPX3 using IHC staining and our data showed a difference in the pattern of GPX3 staining between normal and WM samples, with WM samples showing strong extracellular staining in the surrounding area of the malignant B-cells whereas, the normal BM displayed a disperse cellular staining, without any evident of GPX3 positivity in the extracellular matrix (Fig. 3B). This data additionally supported our previous findings that GPX activity is upregulated in WM tumor microenvironment. It is known that GPX activity protects cells against oxidative stress by reducing lipid hydroperoxides and hydrogen peroxide (H2O2) and conversion of glutathione to glutathione disulfide [20]. This result implies that increased GPX activity is a mechanism to counteract the effects of elevated reactive oxygen species (ROS) that are produced in the course of tumor cell growth. To discover the significance of GPX3 in promoting WM cell survival, we knocked down GPX3 gene expression using siRNA and analyzed viability of the BCWM.1 cells. The transfection efficiency of the cells were about 40% (data is not shown). GPX3 siRNA significantly (p < 0.01) reduced viability of the cells (Fig. 3C and Fig. 3D), validating our proposition that decreased GPX3 expression could decrease the survival of the cells.

The cytokine composition of the tumor microenvironment increases glutathione synthesis in WM.

**Fig. 4:**
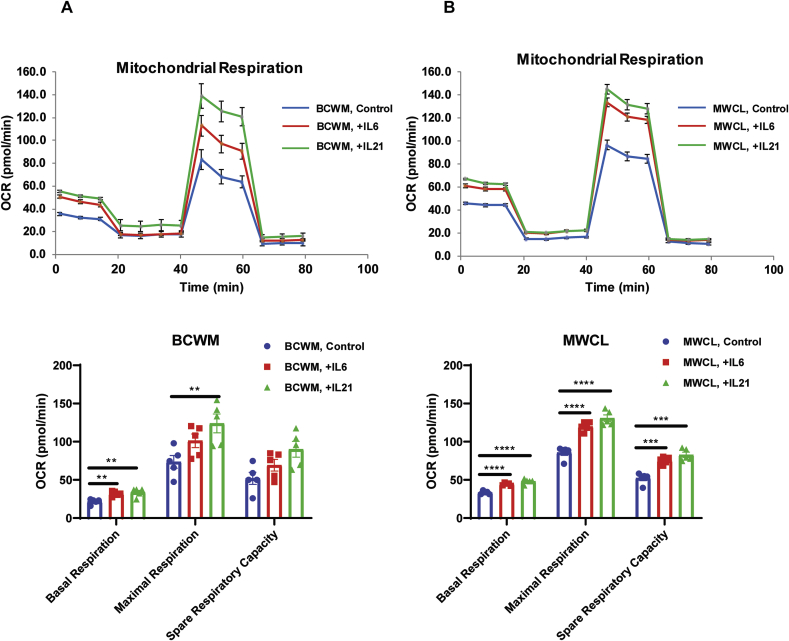
Cytokines IL-6 and IL-21 increase oxygen consumption rate in WM. Mitochondrial stress test analysis shows the effect of IL-6 or IL-21 on the oxygen consumption rate (OCR) in both BCWM.1 **(A)** and MWCL-1 **(B)** cell lines using Agilent XFe96 seahorse analyzer. Data is the mean ± SE of five replicates (n = 5). Bar graphs compare the basal respiration, maximal respiration and spare respiratory capacity between untreated and treated groups. Significant differences are shown as **p < 0.01, ***p < 0.001, ****p < 0.0001 on the bar graphs.

It has been demonstrated that IL-6 and IL-21 in the WM tumor microenvironment, induce tumor cell proliferation and IgM secretion [21,22]. To define a role for these cytokines in modulating redox status in WM, we first assessed the mitochondrial respiratory capacity in the WM cells since the mitochondrial respiratory chain is known as the main source of ROS generation [23]. Treatment with IL-6 or IL-21 increased the mitochondrial respiration (Fig. 4A and Fig. 4B) including basal respiration, maximal respiration and spare respiratory capacity, implying that the increased levels of IL-6 and IL-21 could be associated with enhanced ROS production.

**Fig. 5:**
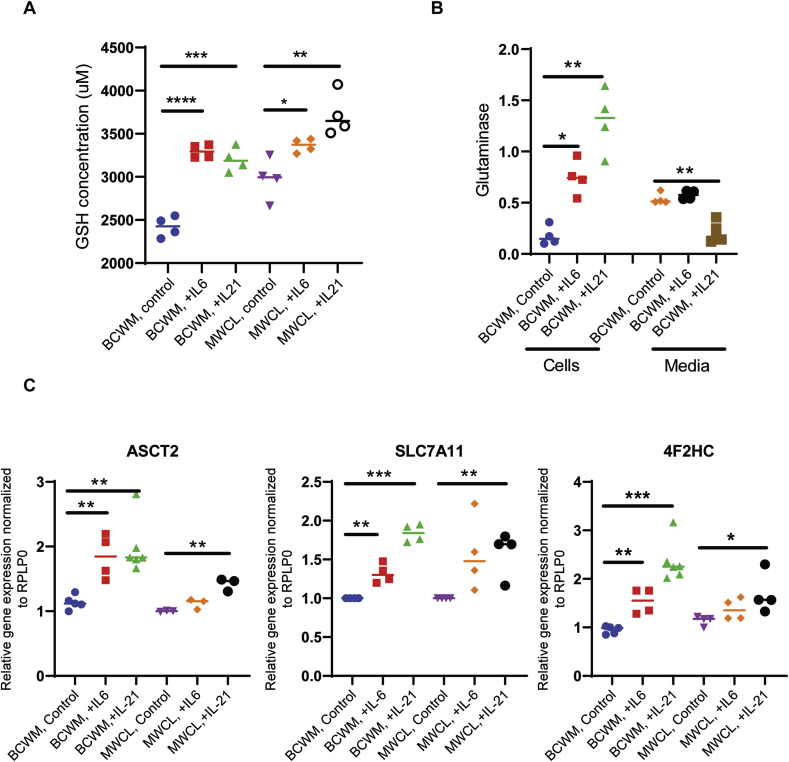
The effect of IL-6 and IL-21 on glutathione metabolism in WM. A) Bar graphs show the reduced glutathione (GSH) level is significantly elevated in the WM cell lines treated with IL-6 or IL-21 (*p = 0.01, **p = 0.005, ***p = 0.0002 and ****p < 0.0001). The data are from four separate experiments. **B)** Bar graphs showing the level of glutaminase in the cell lysates as well as cell culture media of BCWM.1 cells (*p < 0.04, **p < 0.01). **C)** RT-PCR analysis demonstrate the effect of IL-6 or IL-21 on the gene expression of ASCT2 (glutamine transporter), SLC7A11 and 4F2HC (components of cystine/glutamate antiporter on the cell membrane) in WM cell lines (*p < 0.05, **p < 0.01, ****p < 0.0001).

We then studied the effect of these cytokines on glutathione synthesis and found that the glutathione levels were upregulated in both WM cell lines in response to IL-6 or IL-21 treatment (p < 0.03) (Fig. 5A). The glutathione synthesis largely relies on availability of its precursors, amino acids. To investigate whether the synthesis or transport of the amino acids are impacted by IL-6 or IL-21, we measured glutaminase in both cell lysates and media of the treated WM cells. Glutaminase is an enzyme that converts glutamine to glutamate, the latter is utilized by GCLC for the synthesis of glutathione [24]. Cytokine treatment increased glutaminase level in the cells, but reduced in the media (Fig. 5B). We then examined cytokine-induced expression of the amino acid transporters that are key in glutathione synthesis, namely ASCT2, SLC7A11 and 4F2HC [25]. SLC7A11, also called cystine/glutamate transporter, is complexed via disulfide bond with 4F2HC and constitutes *X*_*c*_^*-*^ transport system that modulates glutathione metabolism, regulating cellular redox status [26]. The amino acid transporter, ASCT2, transports the glutamine in an Na+-dependent manner [27]. We found that IL-6 or IL-21 increased the gene expression of ASCT2, SLC7A11 and 4F2HC genes in both cell lines (Fig. 5C). The increased expression of SLC7A11 by WM cells in response to IL-21 treatment was also observed at protein expression level (data not shown). IL-21 treatment also increased glutamate-cysteine ligase regulatory Subunit (GCLm) in WM cells, the effect that was more prominent in BCWM.1 cells rather than BCWM.1 cell lines (Supplementary Fig. 1B). Altogether, these data indicated that increased IL-6 and IL-21 levels in could contribute to increased glutathione metabolism in WM.

Inhibition of glutathione synthesis reduces WM cell proliferation both *in vitro* and *in vivo* xenograft murine models.

**Fig. 6:**
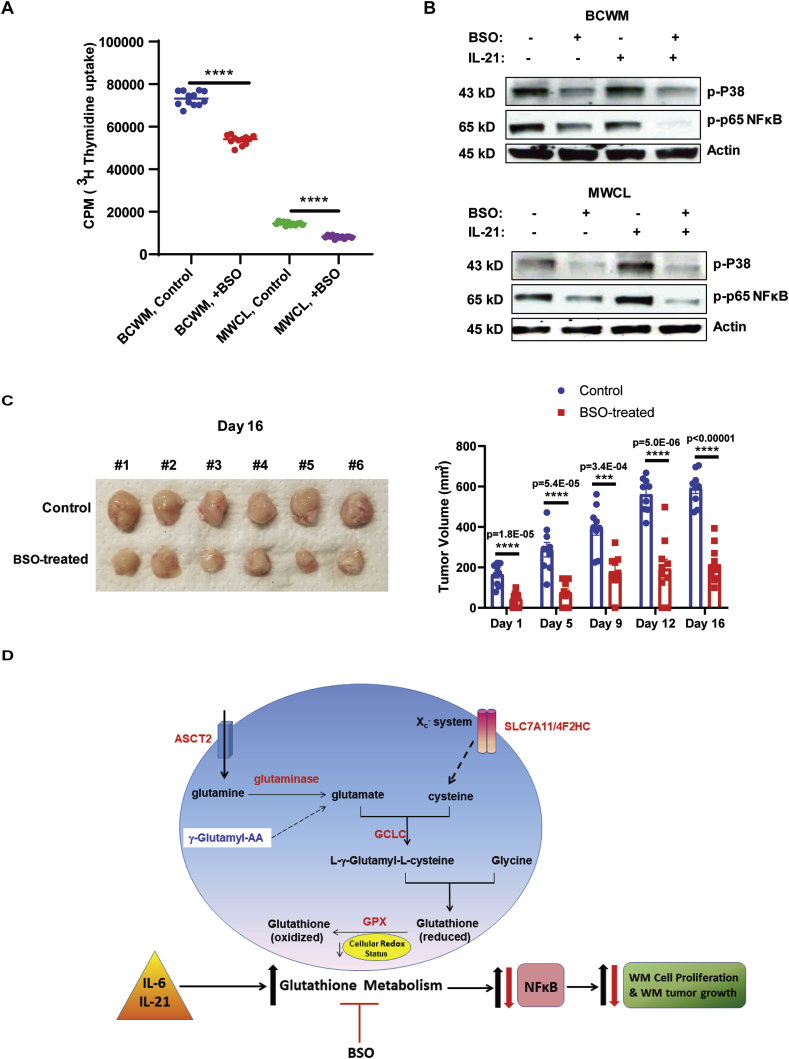
Inhibition of glutathione synthesis reduces the proliferation of WM cells both *in vitro* and *in vivo* murine xenograft tumors. A) Proliferation assay showing the effects of BSO on proliferation of BCWM.1 and MWCL-1 cell lines three days post-treatment using ^3^H-Thymidine incorporation assay. B) Western Blot analysis showing the phosphorylation of NFκB-p65 and MAPK-p38 in the WM cells treated with/without IL-21 (10 μg/ml) and BSO (150 μg/ml). C) Mice were implanted with 4x10^6^ MWCL-1 cells and then treated with 20 mM BSO or left untreated. The volume of the tumors were recorded twice/week after the visible tumor appeared (day0). Tumor growth rate was graphed in both control non-treated and BSO-treated mice over 21 days. D) Schematic representation showing the summary of the data and describes how the increased glutathione metabolism promotes WM cell proliferation (blue arrow) and treatment with BSO reverses this effect (red arrow). (For interpretation of the references to colour in this figure legend, the reader is referred to the Web version of this article.)

Our data showing increased glutathione synthesis and metabolism in WM could explain the mechanism by which WM cells are protected from the harmful effects of increased ROS. Therefore, interference with this pathway could reduce the tumor proliferation in WM. We tested this hypothesis first in an *in vitro*-based assay using Buthionine sulphoximine (BSO), an inhibitor of GCLC enzyme [28]. Our results showed a significant reduction in the number of proliferating WM cells in response to BSO (Fig. 6A). We also examined the secretion of IgM by WM cell lines in response to BSO treatment. Interestingly, BSO treatment for 24 h significantly reduced IgM secretion by BCWM.1 cells. However, IgM secretion by BCWM.1 cells was not affected in the presence of BSO (Supplementary Fig. 1A). To understand the mechanism by which BSO could mediate the suppression of cell proliferation, we measured the phosphorylation of both MAPK-p38 and NFκB-p65 signaling molecules. Increased activation of NFκB has previously been reported to mediate WM cell proliferation in both MyD88^mut^ and MyD88^wt^ WM [29,30]. As shown in Fig. 6B, BSO reduced phosphorylation of p65 and p38 in both control and IL-21 treated cells, suggesting that the effect of glutathione metabolism on WM cell proliferation is mediated in a p38-and NFκB-dependent manner. Interestingly, the reduction in p65 phosphorylation was more prominent in IL-21 treated cells, indicating that BSO could effectively impede the effect of IL-21 in WM cells.

To study the effect of BSO on tumor growth *in vivo*, NSG mice were implanted with WM cells and treated with 20 mM BSO, the dose that effectively depletes glutathione and has no toxicity to the mice [31,32]. BSO-treated mice showed significant reduction in the tumor growth rate (Fig. 6C) compared to the control mice, highlighting that inhibition of glutathione synthesis could suppress WM tumor growth.

## 4 Discussion

The current therapeutic approaches for WM, which encompass BTK-inhibitor, proteasome inhibitor-, chemotherapeutic- and/or rituximab-based therapies [16], have been associated with increased survival rate of the WM patients. However, certain patients remain refractory or develop post-therapy resistance, indicating a need to identify novel targetable molecules. Thus, the molecular mechanisms explaining the pathogenesis of WM have been the focus of many studies over the past decade. The presence of genetic aberrations, including somatic mutations in MyD88 (>90%), CXCR4 (30–40%) and ARID1A (17%) genes in the LPL cells [14] together with changes in microenvironmental factors [21,22] have previously shown as the key players in WM disease characteristics. In an attempt to identify novel molecules modulating WM pathogenesis, we employed a comprehensive metabolomics analysis in the serum and BM plasma of the WM patients and normal donors and identified a total of 75 differentially expressed metabolites. Given that the cancer cells adapt their metabolism to meet their energy requirements [33], the metabolic signature of the WM samples mirrors the presence of the proliferative WM tumor cells. Despite having some common metabolic features, the presence and magnitude of any change in a given metabolite could also be specific to a disease characteristic and therefore serve as a prognostic biomarker. Here, we identified the highest number of the significantly altered metabolites belonged to the metabolism of glutathione. This finding was further corroborated by the untargeted proteomics analysis showing the elevated levels of the enzymes involved in glutathione metabolism. Glutathione is a tripeptide that removes ROS [34] and its increased metabolism in WM is an indication of ongoing oxidative stress condition. Glutathione is a free radical scavenging system that counteracts the detrimental effects of ROS in the cells with high tumorigenic capacity, and the pharmacological inhibition of this system can induce radiosensitization in cancer cells [35]. Several previous studies have explored the role of oxidative stress in hematological malignancies. Depletion of glutathione by BSO or inhibition of *Xc*^−^ system is shown to potentiate the bortezomib cytotoxicity in myeloma cells and overcomes the bortezomib resistance in MM [36,37], highlighting the link between oxidative stress and MM. Similarly, in DLBCL, increased expression of GPX4 gives rise resistance to ROS-induced cell death and is associated with poor outcome in the patients, indicating that malignant cells turn on the antioxidant mechanism to survive [38]. High levels of glutathione-S-Transferase-π (GST-π) have been detected in mantle cell lymphoma (MCL) and its increased expression is believed to contribute to alkylating agents and anthracycline resistance in this disease [39,40]. In agreement with this report, plasma concentration of GST-π is higher in advanced stages of the disease and act as a prognostic factor in non-Hodgkin lymphoma [41]. Therefore, elevated oxidative stress and glutathione metabolism in WM suggest that the manipulation of cellular redox system in WM tumor cells could make them vulnerable to the cell death. The use of alkylating agent ‘Bendamustine’ in combination with ‘Rituximab’ (BR) is shown as an effective therapy for the treatment of WM patients with relapsed/refractory disease [42] and also in newly diagnosed patients [43]. However, the lack of prolonged response, particularly in relapsed/refractory patients, could imply the detoxification mechanism employed by increased glutathione metabolism in WM, highlighting that the combination treatment with BR and small-molecule inhibitors of glutathione synthesis could benefit the patients and prolong the survival in WM.

In this study, we showed the increased total GPX activity in the WM serum and also the upregulation of GPX3 in both WM serum and BM sections. GPX3 staining was more predominant at extracellular spaces, underlining its protective role against ROS effects. We further validated the significance of GPX3 in promoting WM cell survival by GPX3 knock-down approach. GPX3 is considered the only secretory protein among GPX family of the proteins [44] and its upregulation promotes the ovarian cancer progression by modulating tumor microenvironment redox condition [45]. Proteomics analysis on the cerebrospinal fluids of the extranodal NK-/T-cell lymphomas patients has identified GPX3 as a predictor of metastatic disease [46]. Hypermethylation of GPX3 has also been reported as a cancer risk factor [47].

In this study, we also provided evidence that IL-6 and IL-21, cytokines that are increased in the WM tumor microenvironment and contribute to the pathobiology of the disease [21,22], increase glutathione and glutaminase levels in addition to elevating gene expression of ASCT2, SLC7A11 and 4F2HC, the amino acid transporters located on the cell membrane. Given the significant role of *X*_*c*_^*-*^ system (SLC7A11 and 4F2HC) in glutathione metabolism, the effect of IL-6 and IL-21 on increased glutathione level could, at least partially, be mediated by increased expression of these molecules. We also explored the significance of glutathione metabolism in promoting WM cell proliferation on a series of *in vitro* studies and extended our observation to an *in vivo* xenograft murine tumor model. Interestingly, our data showed that inhibition of glutathione synthesis using BSO significantly reduced the proliferation of the WM cells, indicating that the tumor cells require glutathione to support their growth and proliferation. Additionally, we found that the inhibition of glutathione synthesis using BSO reduces phosphorylation of NFκB-p65 and MAPK-p38. In MyD88^mut^ WM, activation of NFκB is induced by BTK and IRAK4/IRAK1 signaling molecules [30]. Instead, the potential activation of NFκB in MyD88^wt^ is attributed to the somatic mutations found in certain genes including *MALT1*, *BCL10*, *NFKB2*, *NFKBIB*, *NFKBIZ,* and *UDRL1F* [29]. Thus, reduced phosphorylation of NFκB-p65 in response to BSO, could suggest that glutathione metabolism and NFκB signaling are linked to each other, regulating the increased proliferation of WM tumor cells. We also detected reduced MAPK-p38 phosphorylation in response to BSO treatment in both control and IL-21 treated WM cell. It has previously been shown that MAPK-p38 activation is induced in response to ROS generation during tumorigenesis [48], so its activation in WM could be a signal of stress response that is linked to glutathione metabolism in WM.

Though we did not explore the effect of specific genetic mutation on glutathione metabolism here, the presence of ARID1A mutation could explain one the reasons for the increased glutathione metabolism in WM patients. A very recent study has reported that ARID1A regulates the glutathione metabolism through an increased transcription of SLC7A11, so that the inhibition of glutathione metabolism increases ROS level and induces apoptosis in cancer cells [49].

Moreover, we found that BSO treatment significantly reduced WM tumor growth rate in the xenograft mice bearing WM tumors, further potentiating our findings that glutathione is a major contributor to tumor growth and proliferation. The inhibition of glutathione has been previously studied in preclinical animal models, showing that BSO is synergistically able to enhance melphalan activity, the chemotherapeutic agents used for MM treatment [50]. Inhibition of glutathione is also shown to overcome the Bcl2-mediated cisplatin resistance in MCF7 breast cancer cells [51]. In this view, the use of pro-oxidants, rather than anti-oxidants, have been promising synergistic based therapies in B-cell malignancies based on the fact that malignant B-cells have increased ROS production [52]. For instance, in DLBCL and primary MCL cells, the combination of BSO with an inhibitor of the thioredoxin system, are able to synergistically reduce glutathione level and induce cell death in an NFκB-dependent manner [53]. Altogether these studies provide convincing evidences that interfering with glutathione metabolism could potentiate the cancer cell-death, in several cancers, including the hematological malignancies.

In summary, our data represents the glutathione anti-oxidant system as the crucial mechanism that promotes WM cell growth and proliferation (Fig. 6D). Treatment strategies targeting glutathione pathway could potentially benefit WM patients. Further investigation is warranted to identify the effect of combination therapies using current treatment regimens and specific inhibitors of the glutathione pathway on the patients with WM.

## Author contributions

SJ designed the experiments, performed the experiments and wrote the manuscript; JS, MS, LEW, JA, HK, XT, ZY helped in some lab works; NA performed the proteomic analysis and analyzed the proteomic dataset. AB supported some bioinformatics analysis; JP involved in reviewing patients database; AN and TEW involved in advising and editing the manuscript; SMA supervised the study and provided scientific inputs. All authors read and revised the manuscript.

## Declaration of competing interest

The authors have no conflict of interest to disclose.

## Figures and Tables

**figs1:**